# A Novel Orf Virus D1701-VrV-Based Dengue Virus (DENV) Vaccine Candidate Expressing HLA-Specific T Cell Epitopes: A Proof-of-Concept Study

**DOI:** 10.3390/biomedicines9121862

**Published:** 2021-12-08

**Authors:** Alena Reguzova, Nico Fischer, Melanie Müller, Ferdinand Salomon, Thomas Jaenisch, Ralf Amann

**Affiliations:** 1 Department of Immunology, Interfaculty Institute for Cell Biology, University of Tübingen, 72076 Tübingen, Germany; alena.reguzova@uni-tuebingen.de (A.R.); melanie.mueller@uni-tuebingen.de (M.M.); ferdinand.salomon@uni-tuebingen.de (F.S.); 2 Department of Infectious Diseases, Heidelberg Institute of Global Health (HIGH) & Tropical Medicine, Heidelberg University Hospital, 69120 Heidelberg, Germany; nico.fischer@fischer-grossvillars.de (N.F.); thomas.jaenisch@uni-heidelberg.de (T.J.)

**Keywords:** parapoxvirus, ORFV, viral vector, dengue virus, vaccine, T cell epitope, HLA class I, immune response, CD8^+^ T cells

## Abstract

Although dengue virus (DENV) affects almost half of the world’s population there are neither preventive treatments nor any long-lasting and protective vaccines available at this time. The complexity of the protective immune response to DENV is still not fully understood. The most advanced vaccine candidates focus specifically on humoral immune responses and the production of virus-neutralizing antibodies. However, results from several recent studies have revealed the protective role of T cells in the immune response to DENV. Hence, in this study, we generated a novel and potent DENV vaccine candidate based on an Orf virus (ORFV, genus *Parapoxvirus*) vector platform engineered to encode five highly conserved or cross-reactive DENV human leukocyte antigen (HLA)-A*02- or HLA-B*07-restricted epitopes as minigenes (ORFV-DENV). We showed that ORFV-DENV facilitates the in vitro priming of CD8^+^ T cells from healthy blood donors based on responses to each of the encoded immunogenic peptides. Moreover, we demonstrated that peripheral blood mononuclear cells isolated from clinically confirmed DENV-positive donors stimulated with ORFV-DENV generate cytotoxic T cell responses to at least three of the expressed DENV peptides. Finally, we showed that ORFV-DENV could activate CD8^+^ T cells isolated from donors who had recovered from Zika virus (ZIKV) infection. ZIKV belongs to the same virus family (*Flaviviridae*) and has epitope sequences that are homologous to those of DENV. We found that highly conserved HLA-B*07-restricted ZIKV and DENV epitopes induced functional CD8^+^ T cell responses in PBMCs isolated from confirmed ZIKV-positive donors. In summary, this proof-of-concept study characterizes a promising new ORFV D1701-VrV-based DENV vaccine candidate that induces broad and functional epitope-specific CD8^+^ T cell responses.

## 1. Introduction

With an estimated 3.9 billion individuals in the population currently at risk, dengue virus (DENV; family *Flaviviridae*) has the potential to affect half of the world’s population in 128 countries [1]. An estimated 390 million DENV infections occur annually, although only ~25% of these infections lead to contact with a health provider [2,3]. Many DENV infections remain undiagnosed because the course of the disease can be asymptomatic in a substantial proportion of affected individuals [4]. In recent decades dengue has spread to nearly all continents, including South America, Africa, Southeast Asia, North America, and, most recently, Europe [5]. The first autochthonous infections have already been reported in France, Croatia, Portugal [6,7,8,9], and in the US [10].

DENV is transmitted by mosquitoes of the species *Aedes aegypti* and *A. albopictus* [5]. These mosquito vectors are expected to spread the disease further to regions that were previously unaffected by DENV [11,12,13,14,15]. Although potential inhibitors of DENV proteins have been identified [16,17,18], there are no specific treatments available for use against DENV at this time. Thus, vector control programs, DENV surveillance studies, and vaccine development for the prevention of this infection are all urgently needed.

At this time, a recombinant, live-attenuated tetravalent dengue vaccine (CYD-TDV), developed and marketed by Sanofi Pasteur, is the only licensed vaccine available to prevent DENV infection [19,20,21]. However, due to safety issues identified in DENV-naïve individuals, this vaccine is recommended only for those who have tested positive for previous DENV infections. Specifically, results obtained from originally seronegative vaccine recipients revealed that they were at higher risk for developing severe dengue upon subsequent encounter with natural infection [19,20]. Two additional late-phase live-attenuated vaccines, the tetravalent dengue vaccine (TDV) candidate TAK-003 from Takeda Pharmaceutical Company and Merck & Co.’s TV003/TV005, have been evaluated in clinical trials [19,20,22,23,24]. TAK-003 showed a vaccine efficacy of 65% against virologically confirmed dengue in seropositive individuals and 54.3% in seronegative individuals throughout three years; however, it showed reduced efficacy against the DENV-3 serotype [25]. While the candidate vaccine from Merck & Co. demonstrated both efficacy and responsive immunogenicity in phase I and phase II clinical trials, vaccinations were associated with significant adverse events [26]. Therefore, uncertainty regarding the generation of long-term protection, the need for booster vaccinations, and the possibility of severe secondary infections remains [19,20,27].

Thus, despite the recent development of several promising candidates, there remains a need for additional vaccines and strategies, most notably those that feature new concepts and that have the potential to elicit both humoral and cellular immune responses [28,29,30]. The increased risk for severe outcomes in DENV-naïve individuals who received the CYD-TDV vaccine demonstrated that the specific immune mechanisms involved in vaccine-associated disease enhancement are not yet well-understood [29,31,32,33]. Several studies have suggested that specific T cell responses may provide control of DENV infections and thus may be essential components of an efficient immune response [30,34,35,36]. Although results from previous human studies implied that the pathogenesis of enhanced disease was associated with the emergence of sub-neutralizing humoral responses and non-specific T cells, results from animal model studies revealed the specific protective role played by CD8^+^ T cells [29,37]. Moreover, human leukocyte antigen (HLA) alleles were recently linked to the differential magnitude of specific CD8^+^ T cell responses and the associated protection from severe forms of acute disease [34]. Of specific note, HLA alleles that correlated with severe DENV disease were those that induced weak CD8^+^ T cell responses. Hence, the identification of robust correlates of protection in natural immunity and responses to vaccination are needed, including the role of critical T cell populations. There are currently no viral-vector-based vaccine candidates that are capable of inducing significant CD8^+^ T cell responses.

We previously reported the development of a new viral vector platform that could be used to promote the expression of heterologous antigens [38] based on the attenuated Orf virus (ORFV) strain D1701-VrV within the family *Poxviridae*. ORFV D1701-VrV is a promising viral vector candidate based on several critical properties, including (i) the very restricted host range of wild-type ORFV (infection causes localized contagious pustular dermatitis in sheep and goats) [39,40], (ii) the lack of systemic spread, (iii) its capacity to induce short-term vector-specific immunity without neutralizing antibodies, (iv) its strong immune-modulating properties, and (v) its ability to induce robust and long-lasting immune responses to vector-encoded heterologous antigens [38,39]. Results from previous studies revealed that ORFV D1701-VrV is well-suited for delivering heterologous viral antigens and generating protective immunity against acute and persistent virus infections that are controlled by both humoral and cellular immune mechanisms [41,42,43].

In the present study, we explore the utility of ORFV D1701-VrV for the development of a vaccine to induce CD8^+^ T cell responses against DENV (ORFV-DENV). We found that ORFV-DENV, expressing conserved HLA-restricted DENV epitopes, demonstrated a high potential for the in vitro priming of naïve CD8^+^ T cells of healthy donors as well as the capacity for specific activation of the memory CD8^+^ T cell repertoire of DENV-seropositive individuals. Furthermore, restimulation assays using highly conserved DENV peptide epitopes also evoked memory CD8^+^ T cell responses in peripheral blood cells isolated from Zika virus (ZIKV)-seropositive individuals. Collectively, our results provide a proof of concept as a first step in the clinical development of an ORFV-DENV vaccine for the induction of HLA-specific CD8^+^ T cells against viral epitopes that are highly conserved among several members of the virus family *Flaviviridae*.

## 2. Materials and Methods

### 2.1. Selection of DENV Peptides

Epitopes were identified using the Immune Epitope Database and Analysis Resource (IEDB). The search included peptides with the following characteristics: (i) linear epitopes (ii) present in at least one of four DENV serotypes that (iii) induce positive responses in either functional T cell assays or HLA-binding affinity assays (iv) restricted to HLA-A*02 or HLA-B*07 alleles (v) in human samples. The pre-defined set of epitopes was further diminished by excluding non-conserved, highly variable peptide sequences between the four DENV serotypes. Finally, the top five epitopes capable of inducing strong cross-reactive CD8^+^ T cell responses between DENV serotypes as well as ones with high sequence conservation were selected.

### 2.2. Cloning of Transfer Plasmids

The genes encoding HLA-B*07- and HLA-A*02-restricted DENV-derived peptides (DENV; CMV-, EBV-, and influenza-A-virus-derived peptides (ReCoA2/B7)), and HIV-1- and ovarial carcinoma (OvCa)-derived peptides (PrCoA2/B7) (synthesized by Gene Art, Thermo Fisher Scientific, Regensburg, Germany) were isolated as a SpeI–HindIII DNA fragment by agarose gel electrophoresis and a Monarch Gel Extraction Kit (New England BioLabs, Frankfurt am Main, Germany) followed by ligation (Quick Ligation Kit, New England BioLabs, Frankfurt am Main, Germany) into SpeI–HindIII-digested transfer plasmid pV12-GFP, resulting in the transfer plasmids pV-DENV-2-GFP, pV-ReCoA2/B7-2-GFP, or pV-PrCoA2/B7-2-GFP, respectively. Correct insertions and sequences were verified by restriction digestion and sequencing (Eurofins Genomics, Ebersberg, Germany).

### 2.3. Transfection

The transfer plasmids pV-DENV-2-GFP, pV-ReCoA2/B7-2-GFP, and pV-PrCoA2/B7-2-GFP were used for the transfection of Vero cells (ATCC No. CRL-1586) infected with D1701-V12-Cherry, as described previously [44,45], using a Nucleofector™ (Lonza, Köln, Germany) and a CLB transfection system (Biozym, Hamburg, Germany) to replace the encoded Cherry gene with DENV-GFP, ReCoA2/B7-GFP, or PrCoA2/B7-GFP.

### 2.4. Generation, Selection, and Propagation of ORFV Recombinants

Vero cells were infected with parental D1701-VrV V12-Cherry and subsequently transfected with the transfer plasmids to enable the homologous recombination of transgenes into the vegf-e locus, as described recently [44]. The resulting ORFV recombinants V-DENV-2-GFP (ORFV-DENV), V-ReCoA2/B7-2-GFP (ORFV-ReCoA2/B7), and V-PrCoA2/B7-2-GFP (ORFV-PrCoA2/B7) were pre-selected by fluorescence-activated cell sorting using BD FACS Jazz™ (Beckton Dickinson, Heidelberg, Germany) [46] followed by limiting dilution in 96-well culture plates [45]. The identification of ORFV recombinants was achieved by polymerase chain reaction using insert- and locus-specific primers. The ORFV recombinants were propagated and purified as previously described [45]. Virus titers were determined by a standard plaque assay [45].

### 2.5. Polymerase Chain Reaction (PCR)

The identification and characterization of ORFV-DENV were assessed by insert- and locus-specific PCR using 2x AmpliTaq Gold polymerase (Thermo Fisher Scientific, Regensburg, Germany), 2x Fast Gene Optima Green polymerase (Nippon, Dueren, Germany) as well as insert-specific and vegf-e-locus-specific primers as described previously ([46]; Table S2). PCR products were separated in a horizontal 1% agarose gel stained with Midori Green (Nippon). PCR products were sequence-verified by Sanger sequencing (Eurofins Genomics).

### 2.6. Peptide Synthesis

Synthetic peptides produced using the 9-fluorenylmethyl-oxycarbonyl (FMOC)/tert-butyl strategy on a Liberty Blue Automated Peptide Synthesizer (CEM, Kamp-Lintfort, Germany) as previously reported [47] were kindly provided by Stefan Stevanovic, Department of Immunology, University of Tübingen, Germany.

### 2.7. Generation of HLA Tetramers

Peptide–HLA class I complexes were generated in-house by UV-mediated ligand exchange [48] followed by multimerization with PE- or APC-conjugated streptavidin (Biolegend, Koblenz, Germany).

### 2.8. PMBCs for CD8^+^ T Cell Assays

For the in vitro priming of CD8^+^ T cells, fresh blood samples from 6 HLA-A*02- and/or HLA-B*07-positive healthy donors were obtained from the Centre for Clinical Transfusion Medicine, University Hospital of Tübingen, Germany.

For in vitro CD8^+^ T cell restimulation and cytotoxicity assays, 58 confirmed DENV-positive patients and 4 confirmed ZIKV-positive patients were identified with the assistance of the Section Clinical Tropical Medicine, University Hospital of Heidelberg, Germany. All donors were diagnosed between 2015 and 2018 with an acute or recently resolved DENV or ZIKV infection that was contracted while traveling in an endemic region. Each DENV-confirmed patient was tested and identified as positive using DENV NS1-, DENV-, ZIKV IgM-, or IgG-specific ELISAs, or DENV- or ZIKV-specific PCR. Blood samples were obtained from 18 participants (13 female and 5 male) with ages ranging from 28 to 70 years. PBMCs were isolated from all blood samples by density gradient centrifugation. For HLA typing, lymphocytes in these samples were stained with anti-HLA-A*02-PE or anti-HLA-B*07-PE monoclonal antibodies (Biolegend, Koblenz, Germany) followed by flow cytometry. If not used immediately, samples were stored at −80 °C.

Informed consent was obtained in accordance with the declaration of Helsinki and the Baden-Württemberg Medical Association’s professional code of conduct. This study was performed according to the guidelines of the local ethics committees (507/2017BO1, Tübingen and S-074/2019, Heidelberg).

### 2.9. DC-Based In Vitro Priming of Naïve CD8^+^ T Cells with ORFV-DENV

CD14^+^ monocytes isolated from PBMCs using CD14 MicroBeads (Miltenyi, Biotec, Bergisch Gladbach, Germany) were seeded into Iscove’s Modified Dulbecco’s Medium (IMDM; Gibco, Thermo Fisher Scientific, Regensburg, Germany) supplemented with 10% of heat-inactivated fetal bovine serum (FBS; Capricorn Scientific GmbH, Ebersdorfergrund, Germany) and 1% penicillin/streptomycin (P/S; Sigma-Aldrich, Steinheim, Germany) in T175 cell culture flasks. These cells were differentiated into DCs by culturing for four days with 86 ng/mL granulocyte-macrophage colony-stimulating factor (Leukine^®^, Sanofi-Aventis Deutschland GmbH, Neu-Isenburg, Germany) and 10 ng/mL interleukin (IL)-4 (Peprotech, Hamburg, Germany). The CD14-negative cell fraction was cultured in T175 flasks in Roswell Park Memorial Institute (RPMI) 1640 medium (Gibco, ThermoFisher Scientific) supplemented with 5% heat-inactivated human AB serum (Sigma, Sigma-Aldrich), 1% P/S, and 5 ng/mL IL-7 (Peprotech, Hamburg, Germany). DCs (105 per well) were seeded into a 96-well cell culture plate (Costar, Corning, Kaiserslautern, Germany) and infected with ORFV-DENV for 6 h at an MOI of 10. CD8^+^ T cells were isolated from the CD14-negative cell fraction using CD8 MicroBeads (Miltenyi Biotec). Infected DCs were co-cultured with 3–8 × 10^5^ CD8^+^ T cells per well and supplemented with 5 ng/mL IL-12 (Promokine, PromoCell GmbH, Heidelberg, Germany). For each well half the medium was exchanged every 3–4 days and supplemented with 40 U/mL IL-2 (R & D Systems, Wiesbaden, Germany). After seven days of co-culturing, the cells were restimulated with irradiated, autologous CD14-negative and CD8-depleted PBMCs loaded with 25 µg/mL of respective synthetic peptides. After four consecutive restimulations, antigen-specific CD8^+^ T cells were evaluated by HLA tetramer staining and/or intracellular cytokine staining (ICS).

### 2.10. Restimulation of Peptide-Specific CD8^+^ T Cells In Vitro with ORFV-DENV

PBMCs (2–3 × 10^6^) from confirmed DENV-positive individuals were seeded into wells of a 24-well culture plate containing 1.5 mL of RPMI 1640 medium supplemented with 5% heat-inactivated human AB serum and 1% P/S. Duplicate samples of PBMCs were infected with ORFV-DENV (MOI 5–30). Synthetic peptides (1 µg/mL) were used for positive controls. PBMCs exposed to ORFV D1701-VrV, encoding GFP only, were used as negative controls. Twenty-four hours after infection, 0.5 mL of the respective media containing 20 U/mL IL-2 was added to each culture and exchanged on days 3, 5, 7, and 9 thereafter. The activation and expansion of antigen-specific CD8^+^ T cells were assessed by HLA tetramer staining and ICS on day 12.

### 2.11. Cytotoxicity Assay

Target JY cells (4 × 10^6^) were labeled with CellTrace Violet or carboxyfluorescein succinimidyl ester (CFSE; ThermoFisher) according to the manufacturer’s instructions and loaded with 30 µg/mL of peptides APTRVVAAEM (Dengue NS31698–1707) or TPRMCTREEF (Dengue NS52881–2890) for 2 h at 37 °C. Negative control cells were loaded with peptide SLYNTVATL (HIV-1 p17 Gag77-85). Controls included PBMCs from ZIKV-positive patients that were restimulated with ORFV-DENV at an MOI of 5, as described above, and with the synthetic peptides APTRVVAAEM (Dengue NS31698–1707) or TPRMCTREEF (Dengue NS52881–2890). Effector and target cells were seeded together in 96-well U-bottom plates (Costar) at an effector-to-target ratio of 0.1:1. After 14 h of incubation, cells were stained with SYTOX Deep Red (ThermoFisher) and analyzed by flow cytometry. Cell-specific lysis was expressed as the percentage of (dead) SYTOX Deep Red-positive cells within the population of CellTrace-Violet- or CFSE-positive target cells after correcting for nonspecific lysis (i.e., lysis in response to SLYNTVATL HIV-1 p17 Gag77-85-loaded target cells).

### 2.12. Flow Cytometric Analysis of Antigen-Specific T Cells

All flow cytometric measurements were performed on a FACS LSR Fortessa (Beckton Dickinson). For HLA tetramer analysis, half of the primed or restimulated T cells from healthy participants or confirmed DENV-positive donors were transferred to 96-well plates and incubated for 20 min at room temperature (RT) with 2.5 mg/mL of the respective tetramers in a solution (in 1× phosphate-buffered saline (PBS); Gibco, ThermoFisher Scientific) supplemented with 0.02% sodium azide (Merck, Darmstadt, Germany), 2 mM ethylenediaminetetraacetic acid (EDTA) (Carl Roth, Karlsruhe, Germany), and 50% FBS (Capricorn Scientific GmbH). Subsequently, live/dead staining was performed with a Zombie Aqua™ Fixable Viability Kit (Biolegend) according to the manufacturer’s instructions. Surface molecules were stained with anti-CD4-PacificBlue, anti-CD8-APC/Cy7, and anti-CD3-FITC (Biolegend) monoclonal antibodies for 30 min at 4 °C.

ICS was performed by incubating half of the in-vitro-primed or restimulated T cells of healthy or confirmed DENV-positive donors with 2 µg/mL synthetic DENV-derived or control peptides and 2 µg/mL brefeldin A (Sigma-Aldrich) for 12 h. Afterward, live/dead staining was performed using a Zombie Aqua™ Fixable Viability Kit according to the manufacturer’s instructions; this was followed by staining with anti-CD4-PacificBlue and anti-CD8-APC/Cy7 monoclonal antibodies for 30 min at 4 °C. Cells were permeabilized using Cytoperm/Cytofix solution (BD Bioscience, Heidelberg, Germany) for 30 min at 4 °C followed by a staining with anti-interferon (IFN)-γ-PE and anti-tumor necrosis factor (TNF)-α-AlexaFluor700 (Biolegend).

### 2.13. Alignment of Proteomes Encoded by Viruses of the Family Flaviviridae

Proteomes of different flavivirus isolates were analyzed using a query of the Protein Database at the National Center for Biotechnology Information (NCBI, Bethesda, MD, USA). We focused on randomly selected endemic countries of Africa (n = 3), Southeast Asia (n = 10), Central and South America (n = 10), and the Western Pacific (n = 4). Three hundred and forty-four fully analyzed proteomes were randomly selected, including isolates of DENV-1 (n = 49), DENV-2 (n = 60), DENV-3 (n = 47), DENV-4 (n = 29), Japanese encephalitis virus (JEV; n = 30), West Nile virus (WNV; n = 15), yellow fever virus (YFV; n = 19), and ZIKV (n = 95). Proteomes were aligned using the Clustal Omega multiple sequence alignment tool [49] of the European Bioinformatics Institute (EBI), European Molecular Biology Laboratory (EMBL, Heidelberg, Germany).

### 2.14. Data Processing and Statistics

Analysis of the flow cytometric data was conducted using FlowJo version 10 (Becton Dickinson). GraphPad Prism 7 (GraphPad Software, San Diego, CA, USA) was used for data presentation. Analysis of the multiple-sequence alignment was performed using Jalview version 2.11.0 [50].

## 3. Results

### 3.1. Selection of DENV Peptides

**Figure 1:**
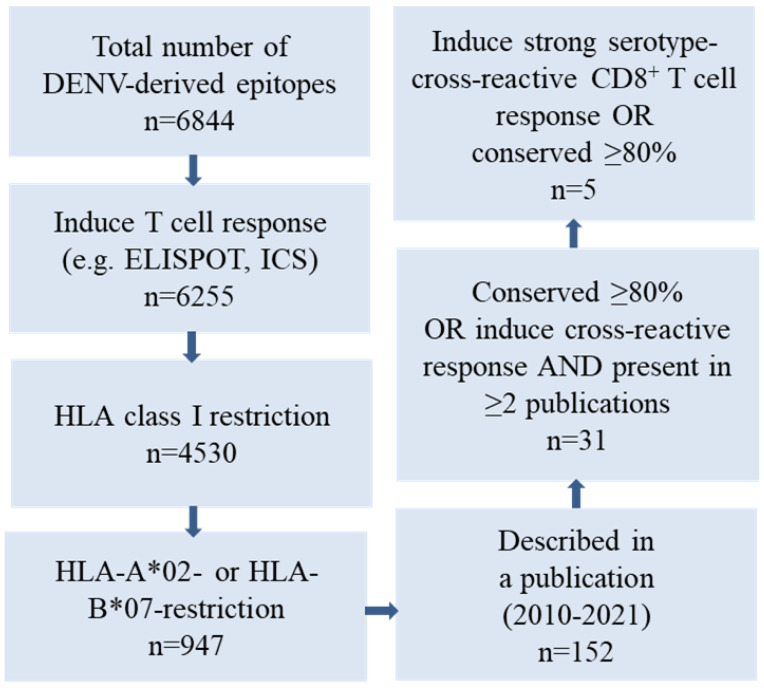
Selection of DENV-derived epitopes based on a query of the Immune Epitope Database (IEDB) of the NIAID. Five DENV-derived HLA-A*02- or HL-B*07-restricted epitopes were selected according to the magnitude of cross-reactive functional CD8^+^ T cell responses reported and/or conservation among the various DENV serotypes.

To generate an ORFV D1701-VrV vaccine candidate, ORFV-DENV, capable of inducing a cellular immune response against several conserved and/or serotype-cross-reactive epitopes of DENV, human leukocyte antigen (HLA)-A*02- and HLA-B*07-restricted immunogenic peptides were selected using the Immune Epitope Database and Analysis Resource (IEDB) of the NIAID [51] (Figure 1). As most of the T cell epitopes cluster within the DENV non-structural (NS) proteins, these regions were explored in depth. Whole-proteome IEDB analysis predicted 6844 DENV epitopes. We excluded epitopes that did not induce positive responses in functional assays, for example, those that did not promote interferon-γ (IFN-γ) synthesis or release as determined by intracellular cytokine staining (ICS) or ELISPOT assays. Our final selection included HLA-A*02- or HLA-B*07-restricted epitopes as determined by HLA competition or tandem mass spectrometry (MS/MS) epitope prediction assays as well as those described in previous publication [30,34,52,53,54,55,56,57,58,59,60,61] (Figure 1).

One hundred and fifty-two peptides that were conserved at ≥80% or exhibited strong multifunctional cross-reactive responses against other serotypes were identified in various publications [30,34,52,53,54,55,56,57,58,59,60,61]. Finally, three HLA-A*02- and two HLA-B*07-restricted epitopes encoded by DENV NS3, NS4B, NS5, and capsid (C) proteins were selected for use in the ORFV-DENV candidate vaccine. HLA binding affinity algorithms in NetMHC 4.0 [62] and the SYFPEITHI database [51] identified four of these peptides as strong HLA binders (NetMHC percentile rank < 0.5, Table 1). The peptide derived from the sequence of the virus NS5 protein, KLAEAIFKL, originally identified in DENV serotype 2, is conserved as its C-terminus has already been identified as a protective epitope in both natural infection and vaccination studies [53,55,57]. The peptides NIQTAINQV and VTLLCLIPTV were both identified by MS/MS epitope prediction; the NIQTAINQV peptide is highly conserved in NS4B proteins of various DENV serotypes [57]. The DENV serotype 4 C protein-derived sequence, VTLLCLIPTV, elicited strong cross-reactive CD8^+^ T cell responses in different virus serotypes [57]. Moreover, in previous vaccine studies, the homologous sequence ITLLCLIPTV, found in the C protein of DENV serotype 2, induced a T cell response in study participants [30]. The NS3 protein epitope, APTRVAAEM, is highly conserved among all viruses of the family *Flaviviridae* and was identified as an essential epitope in both natural infection and vaccination studies; the same has been reported for the NS5-derived peptide, TPRMCTREEF [30,34,52,54,55]. Both HLA-B*07-restricted peptides are predicted to have strong binding affinities to the HLA class I complex (Table 1).

### 3.2. Generation of ORFV-DENV and Control Recombinants

**Figure 2:**
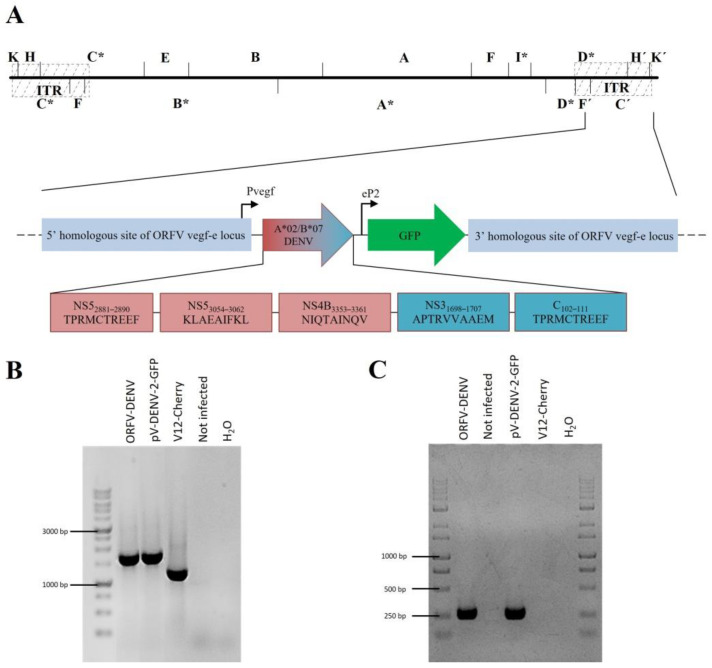
(**A**) Genomic map of ORFV-DENV depicting the arrangement of the minigenes encoding HLA-A*02- (red boxes) and HLA-B*07- (blue boxes) restricted DENV-derived epitopes controlled by the authentic early promoter Pvegf. The GFP gene used as a selection marker for virus purification is expressed under the control of the synthetic eP2 promoter. The 5′ and 3′ sequences homologous to the vegf-e locus of ORFV flanked the minigenes and the GFP sequence, enabling direct homologous recombination with the virus backbone. * DNA fragments affected by the genomic deletions (**B**) The genetic homogeneity of novel recombinants was verified by PCR targeting the vegf-e locus and resulted in the predicted amplicon size for ORFV-DENV (1640 bp). (**C**) DENV-specific PCR revealed the predicted amplicon sizes for ORFV-DENV (277 bp). Transfer plasmid pV-DENV-2-GFP was used as the positive control. DNA isolated from the parental ORFV V12-Cherry and non-infected cells served as the negative control.

In total, three ORFV recombinants were generated. ORFV-DENV encodes the three HLA-A*02- and two HLA-B*07-restricted DENV peptides in a minigene format (Table 1, Figure 2A). ORFV-ReCoA2/B7 encodes three HLA-A*02-restricted cytomegalovirus (CMV; human herpesvirus (HHV) 5), Epstein–Barr Virus (EBV; HHV-4), and influenza A virus (IAV) epitopes together with two HLA-B*07-restricted CMV and EBV peptides (Table S1, Figure S1) and served as a control for peripheral blood mononuclear cell (PBMC) restimulation assays. ORFV-PrCoA2/B7 encodes two HLA-B*07-restricted human immunodeficiency virus 1 (HIV-1)- and ovarian cancer (OvCa)-derived peptides and one HLA-A*02-restricted HIV-1 peptide in a minigene format (Table S1, Figure S1), and served as a control for the CD8^+^ T cell priming assay.

The integration of the transgenes was achieved by homologous recombination into the vegf-e locus, as described in Materials and Methods. Recombinant ORFVs were selected for GFP expression, single plaque purified by five consecutive rounds, and correct transgene insertion was verified by vegf-e- (Figure 2B) transgene-specific PCR (Figure 2C) and subsequent gene sequence analysis.

### 3.3. ORFV-DENV Efficiently Primes CD8^+^ T Cells Specific to DENV-Derived Epitopes In Vitro

To evaluate the immunogenicity of ORFV-DENV, a DC-based in vitro priming assay was performed. CD8^+^ T cells were co-cultured with autologous ORFV-DENV-infected DCs from six HLA-A*02- and/or HLA-B*07-positive healthy blood donors. Within the PBMCs, only the CD14^+^ monocytes and antigen-presenting monocyte-derived DCs, but not CD19^+^ B cells, CD56^+^ NK cells, CD4^+^ T cells, or CD8^+^ T cells, were susceptible after exposure to ORFV-DENV (data not shown). CD8^+^ T cell priming was monitored by HLA tetramer staining and ICS.

**Figure 3:**
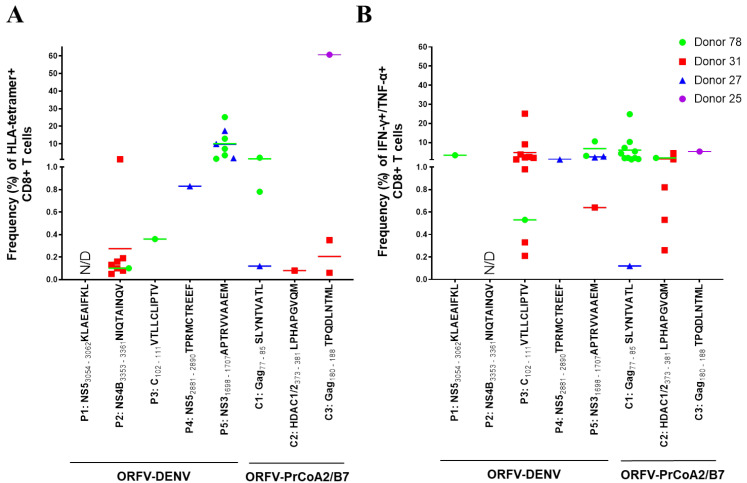
ORFV-DENV induces CD8^+^ T cells against several DENV-derived epitopes within the same individual in vitro. The responses of each donor are identified with different symbols and colors, as shown. Each symbol corresponds to a biological replicate exhibiting a positive response out of thirty (Donors 78, 27, and 25) or twenty (Donor 31) replicates. Effective priming was verified by (**A**) HLA tetramer staining and/or (**B**) ICS. Data for only four donors are shown. Cells from a fifth donor (data included in Table 2 only) showed a cytokine response to a pool of HLA-A*02-restricted DENV and control peptides; the specificity of the observed responses against individual peptides was not determined due to rapid cell death. CD8^+^ T cells from a sixth donor were not primed against any of DENV-derived or control epitopes (data not included). N/D, not determined (negative).

Each DENV-derived epitope elicited a peptide-specific CD8^+^ T cell response as determined by HLA tetramer staining and/or ICS in at least one donor (i.e., 1 of 5, or 20%; Figure 3 and Figure S2, Table 2). The HLA-A*02-restricted DENV NS5 peptide KLAEAIFKL induced a specific CD8^+^ T cell response in one of the five donors evaluated (Donor 78; Figure 3B). Although we detected no peptide-specific CD8^+^ T cells in the tetramer staining experiments (Figure 3A), 3.35% of the CD8^+^ T cells from this donor expressed both TNF-α and IFN-γ in response to KLAEAIFKL as determined by ICS (Figure 3B). The DENV NS4B peptide, NIQTAINQV, induced peptide-specific CD8^+^ T cells in two donors (Donor 78 and Donor 31); HLA tetramer staining revealed overall frequencies of peptide-specific CD8^+^ T cells ranging from 0.05% to 1.21% (Figure 3A). However, many of these positive cells did not produce TNF-α or IFN-γ (Figure 3B). The DENV capsid (C) peptide VTLLCLIPTV also induced peptide-specific CD8^+^ T cell responses in these two donors. While only 0.36% of the CD8^+^ T cells were detected using an HLA tetramer staining assay (Donor 78 only; Figure 3A), ICS used to detect TNF-α and IFN-γ expression revealed CD8^+^ T cell responses in the range of 0.21% to 25.1% (Figure 3B).

HLA-B*07-restricted DENV epitopes induced higher frequencies of peptide-specific CD8^+^ T cell responses compared to those observed in response to HLA-A*02-restricted epitopes (Figure 3). Among these findings, 0.83% of the CD8^+^ T cells from Donor 27 expressing a receptor for the NS5 peptide TPRMCTREEF were detected in the HLA tetramer staining assay (Figure 3A), while the results of ICS indicated that 1.1% of these CD8^+^ T cells expressed both TNF-α and IFN-γ (Figure 3B). The DENV NS3 peptide, APTRVVAAEM, was the most effective in these assays; responses were detected in three of the five healthy donors. Priming with this epitope induced peptide-specific CD8^+^ T cell frequencies as high as 25.1% in the HLA tetramer staining assay (Figure 3A). Notably, 0.64% to 10.6% of these CD8^+^ T cells were also TNF-α- and IFN-γ-positive (Figure 3B).

Furthermore, we note that in vitro priming with ORFV-DENV, encoding all five HLA-A*02- and HLA-B*07-restricted DENV epitopes, led to CD8^+^ T cell responses against several epitopes simultaneously in four of six donors (Figure 3). Notably, CD8^+^ T cells from Donor 78 responded to four of the five DENV-derived epitopes. As shown in Figure 3A, T cells from this donor could be stimulated with the three distinct HLA-A*02-restricted DENV epitopes as well as with the HLA-B*07-restricted peptide, APTRVVAAEM. Peptide-specific responses from CD8^+^ T cells from Donor 31 were detected after priming with two of the HLA-A*02-restricted epitopes as well as to the HLA-B*07-restricted peptide, APTRVVAAEM. CD8^+^ T cells from Donor 27 responded to the HLA-B*07-restricted DENV epitopes only. CD8^+^ T cells from Donor 25 did not respond to any of the DENV-derived epitopes, although cells showed HIV-1-epitope-specific CD8^+^ T cell responses after priming with the control vector. CD8^+^ T cells from one donor responded to a pool of HLA-A*02-restricted DENV and control epitopes; however, detailed analysis of antigen specificities could not be performed due to problems with cell cultivation. CD8+ T cells from a sixth donor did not respond to any of the DENV-derived or control epitopes (not included in the analysis).

Collectively, these data suggest that ORFV-DENV, encoding HLA-class-I-restricted DENV epitopes, can prime naïve CD8^+^ T cells from healthy blood donors and generate responses to all five of the encoded DENV-derived epitopes.

### 3.4. CD8^+^ T Cells Primed with HLA-Class-I-Restricted DENV-Derived Epitopes during Natural Infection Are Activated by ORFV-DENV

Next, we determined whether ORFV D1701-VrV, encoding HLA-A*02- and HLA-B*07-restricted DENV epitopes, could activate memory CD8^+^ T cells from donors who had recovered from a natural infection. For these experiments, PBMCs were isolated from HLA-A*02- and/or HLA-B*07-positive donors who were laboratory-confirmed as DENV-positive. PMBCs from these donors were restimulated with ORFV-DENV in vitro (Table 3, Figure S3). The activation of memory CD8^+^ T cells after restimulation was evaluated by HLA tetramer staining and ICS. Before stimulation, no memory CD8^+^ T cells against any of the encoded DENV peptides were detected in any of these donors, most likely due to their low frequency or absence in peripheral blood.

**Figure 4:**
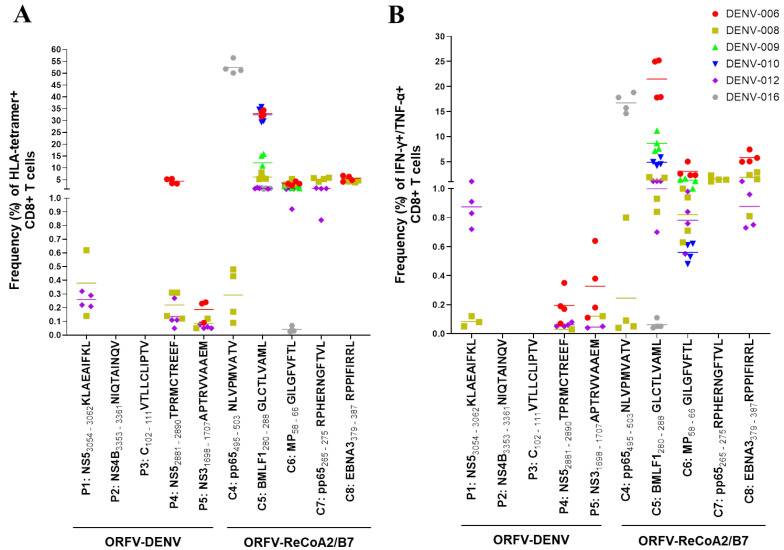
Restimulation of CD8^+^ T cells from six confirmed DENV-positive donors with ORFV-DENV. The responses of each donor are identified with a different symbol and color, as shown. Each symbol corresponds to a biological replicate exhibiting a positive response. Specific CD8^+^ T cell activation was verified by (**A**) HLA tetramer staining and/or (**B**) ICS. PBMCs from donor DENV-015 showed no specific CD8^+^ T cell responses against any of DENV-derived or control epitopes after restimulation (data not shown).

We identified functional CD8^+^ T cells that could be activated with three DENV-derived epitopes in three of six donors. An example of a positive response after restimulation is shown in Figure S4. After restimulation with ORFV-DENV, specific CD8^+^ T cell responses against the HLA-A*02-restricted DENV NS5 epitope KLAEAIFKL were detected in two of the donors with frequencies that ranged from 0.02% to 0.62% using HLA tetramer staining. Comparatively low numbers of functional CD8+ T cells were detected using ICS (Figure 4).

The remaining HLA-A*02-restricted DENV epitopes encoded by ORFV-DENV induced no specific CD8^+^ T cell responses. The tested donors most likely had no memory CD8^+^ T cells that were specific for these peptides. In contrast, both HLA-B*07-restricted DENV-derived epitopes encoded by ORFV-DENV (TPRMCTREEF and APTRVVAAEM) induced the expansion of peptide-specific CD8^+^ T cells. The NS5 peptide TPRMCTREEF induced peptide-specific CD8^+^ T cell frequencies ranging from 0.05% to 5.42%, while up to 0.23% of the CD8^+^ T cells responded to APTRVVAAEM. The functionality of both of these peptide-specific memory T cell populations was demonstrated by ICS (Figure 4). Restimulation with a mock virus that encoded GFP only resulted in no induction of CD8^+^ T cell responses in any of these donors (Figure S4).

Furthermore, the proliferation of peptide-specific CD8^+^ T cells from each donor could be induced by at least two different peptides. Restimulated CD8^+^ T cells from donors DENV-012 and DENV-008 responded to both HLA-B*07-restricted DENV epitopes as well as to the HLA-A*02-restricted NS5 peptide KLAEAIFKL. In contrast, CD8^+^ T cells from donor DENV-006 responded to the HLA-B*07-restricted peptides only (Figure 4).

Interestingly, CD8^+^ T cells were successfully restimulated with DENV-derived epitopes only in the donors that expressed both HLA-A*02 and HLA-B*07 alleles. In contrast, CD8^+^ T cells from donors carrying only HLA-A*02 alleles showed no specific responses to any of the encoded DENV epitopes, although they did respond to the control peptides (Table 3 and Figure 4). CD8^+^ T cells from two donors responded to two HLA-A*02-restricted control peptides, while one donor maintained T cell populations that responded to three of these peptides (Figure 4). Of note, PBMCs from one donor most likely contained no specific CD8^+^ T cells capable of responding to any of DENV-derived or control epitopes after restimulation (data not shown).

Taken together, our results suggest that ORFV-DENV, encoding DENV-derived epitopes, can induce the restimulation of functional memory CD8^+^ T cells in individuals confirmed as DENV-positive.

### 3.5. ORFV-DENV Activates Cross-Reactivate CD8^+^ T Cells Specific for Homologous Epitopes in ZIKV

Flaviviruses share a high degree of gene sequence homology, most notably in the coding sequences of their non-structural (NS) proteins. This observation offers the possibility of generating protective cross-reactive memory CD8^+^ T cell responses using peptides that are highly conserved within this virus family. We assessed this possibility by examining whether recombinant ORFV D1701-VrV, encoding DENV-derived epitopes, could restimulate CD8^+^ T cells that recognize existing ZIKV epitopes in experimental studies carried out in vitro.

First, the proteomes of various DENV serotypes were aligned with sequences of the NCBI reference ZIKV strain MR766 (Table 4). Four of the five DENV-derived peptides expressed by ORFV-DENV showed sequence conservation with analogous peptides in ZIKV within a range of 44% to 100%. As shown, the DENV NS5 peptide KLAEAIFKL and NS4B peptide NIQTAINQV were 44% conserved with analogous peptides from ZIKV, while the HLA-B*07-restricted DENV NS5 and NS3 peptides, TPRMCTREEF and APTRVVAAEM, showed 70% and 100% amino acid sequence conservation with analogous ZIKV peptides, respectively. By contrast, the DENV capsid (C) peptide sequence VTLLCLIPTV was not conserved.

**Figure 5:**
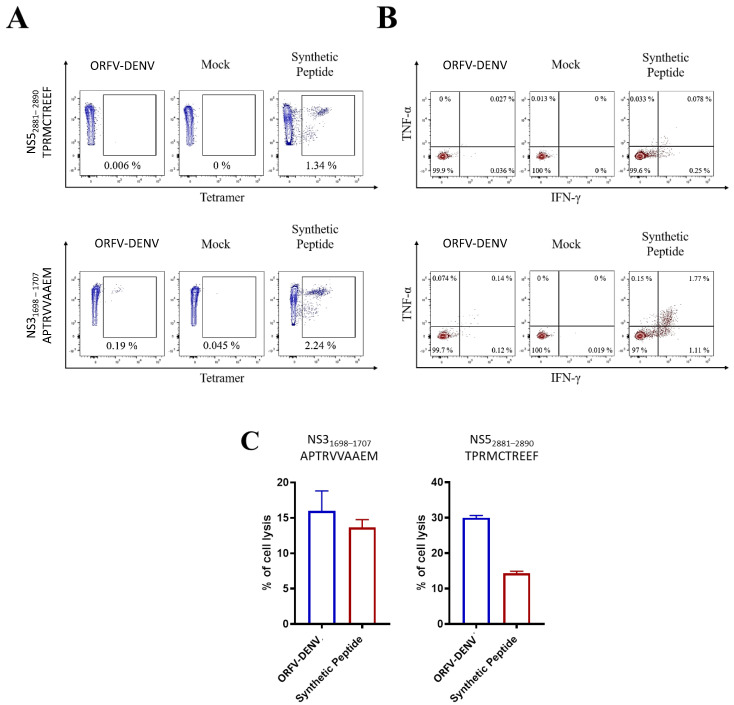
Cross-reactive CD8^+^ T cell responses in PBMCs from confirmed ZIKV-positive donor DENV-021 after in vitro restimulation with ORFV-DENV expressing DENV-derived peptide epitopes. Frequencies and functionality of epitope-specific CD8^+^ T cells were determined by (**A**) HLA tetramer staining, (**B**) ICS, and (**C**) specific lysis after 20 h of co-incubation with peptide-pulsed target cells at an effector-to-target ratio of 0.1:1. The percentage of target cell lysis corrected for spontaneous background lysis is shown. A synthetic peptide was used as a control.

In vitro restimulation of PBMCs from two donors confirmed as ZIKV- (but not DENV-) positive was performed using ORFV-DENV expressing DENV epitopes (Table 3, Figure 4). The activation and functional characterization of cross-reactive CD8^+^ T cells were confirmed by HLA tetramer staining, ICS, and antigen-specific target cell killing assays. No specific CD8^+^ T cell responses were observed in PBMCs from either of the two ZIKV-positive to HLA-A*02-restricted DENV-derived peptides (data not shown). In contrast, cross-reactive CD8^+^ T cells were activated in response to DENV-derived peptides, TPRMCTREEF and APTRVVAAEM, in the one HLA-B*07-positive donor (Table 3, Figure 5). Frequencies measured by HLA tetramer staining indicated that 0.19% of CD8^+^ T cells were specific for the APTRVVAAEM peptide (Figure 5A). Further analysis by ICS revealed that 0.14% of these CD8^+^ T cells expressed both IFN-γ and TNF-α (Figure 5B). Moreover, 18.5% of the ORFV-DENV-restimulated CD8+ T cells were capable of specifically killing APTRVVAAEM-peptide-loaded target cells (Figure 5C).

The HLA-B*07-restricted DENV-derived peptide, TPRMCTREEF, also activated cross-reactive cytotoxic CD8^+^ T cells in a ZIKV-positive donor (Table 3, Figure 5C). After the restimulation of PBMCs with ORFV-DENV, 37.9% of the CD8^+^ T cells were capable of antigen-specific target cell killing (Figure 5C). However, these cells were not detected using either HLA tetramer staining or ICS (Figure 5A,B). Thus, our findings suggest that ORFV-DENV, encoding the HLA-B*07-restricted DENV-derived peptides TPRMCTREEF and APTRVVAAEM, can cross-reactivate and induce functional CD8^+^ T cell immune responses against homologous epitopes in ZIKV.

An alignment of 344 proteomes from various viruses of the family of *Flaviviridae* revealed that the HLA-B*07-restricted DENV NS3 peptide APTRVVAAEM and the HLA-B*07-restricted DENV NS5 peptide TPRMCTREEF are highly conserved, ranging from 70% to 100% amino acid sequence identity. In contrast, the HLA-A*02-restricted DENV peptides including KLAEAIFKL were conserved from 44.4–88.8% among viruses that include ZIKV, DENV, yellow fever virus (YFV), and West Nile virus (WNV). Conservation of the NS4B peptide NIQTAINQV ranged from 22.2–88.8%.

Collectively, these results suggest that the highly conserved, HLA-B*07-restricted DENV-derived peptides TPRMCTREEF and APTRVVAAEM encoded in ORFV-DENV may induce cross-protective immunity against several viruses of the family *Flaviviridae*.

## 4. Discussion

Almost half of the world’s population currently resides in DENV-endemic tropical and subtropical regions. As a result, DENV has become one of the most important arthropod-transmitted viral infections worldwide. The morbidity associated with dengue goes beyond the clinical impact of the disease itself and also includes the resulting socio-economic repercussions that emerge in affected low-income nations [63,64,65]. The global costs for the prevention and treatment of DENV disease are estimated at 8.9 to 39.9 billion US dollars per year [66,67]. Furthermore, climate change, increasing mobility, and the globalization of trade have all accelerated the spread of mosquito vectors. Taken together, these factors translate into future projections of worldwide increases in at-risk populations [5,11,12,13,15,68,69]. Improved surveillance, vector control strategies, treatment options, and the development of safe and effective vaccines are all urgently needed [70,71].

A major challenge to the development of an effective vaccine is our limited understanding of the mechanisms underlying protective immune responses to DENV [72,73,74,75,76]. The three most advanced vaccine candidates, CYD-TDV (Sanofi Pasteur), TDV (Takeda), and TV003/TV005 (Merck & Co.) were designed primarily to activate humoral immunity and induce neutralizing antibodies (nAbs) [19,20]. However, recent findings documenting disease enhancement in a cohort of DENV-naïve vaccinees suggested that humoral responses, including the development of nAbs, may not be strong predictors of vaccine efficacy and may even increase the risk of severe disease [19,74,77,78]. At the same time, the role of neutralizing antibodies and the plaque reduction neutralization test (PRNT), which has been a proxy standard of protection, has been questioned [79].

In contrast, several studies have demonstrated the beneficial role of multifunctional T cell responses in promoting the clearance of DENV and protection against infection [34,60,80]. In heterologous, second natural DENV infections, protective cellular immune responses were traced to highly serotype-conserved epitopes [30]. Vaccination with TDV or TV003/TV005 induces more effective T cell responses than were observed in responses to CYD-TDV. However, the cellular responses induced by TDV were mainly serotype-2-specific and were only partially effective against other serotypes [81]. Most T cell epitopes are clustered within the DENV NS proteins [82]; this observation was utilized in TDV only with respect to the DENV-2 backbone. Another advanced candidate, the tetravalent TV003/TV005 vaccine, induced broad and multifunctional CD8^+^ T cell responses against serotype-conserved NS protein epitopes in addition to a humoral response that resulted in nAbs [30]. However, this vaccine was associated with frequent adverse events and low-level viremia in several clinical studies [19]. These findings clearly outline the clinical importance of vaccine-induced T cell responses against serotype-conserved DENV epitopes. Hence, novel vaccine strategies and candidates that focus on T cell responses may be needed to achieve potent and long-lasting immune responses that involve fewer immunizations, improved safety and compatibility profiles, and reduced costs associated with vaccine production.

Previously, we reported our findings featuring the parapoxvirus, ORFV, as a new viral vector platform. Our ORFV viral vector combines several favorable properties that serve to promote the development of efficient and effective vaccines [38,39,40,44]. Several studies carried out in animal models successfully demonstrated the induction of a strong and long-lasting cellular and humoral immunity, most notably seroconversion against recombinant ORFV-D1701-VrV-encoded heterologous genes [42,83,84,85]. In contrast to the currently available and licensed CYD-TDV vaccine, ORFV D1701-VrV is not a flavivirus and thus it does not elicit sub-neutralizing immune responses. We believe that vaccination with recombinant ORFV D1701-VrV engineered to express selected virus-derived antigens may induce specific immune responses against highly immunogenic and serotype-conserved or cross-reactive T and B cell epitopes.

For this study we developed ORFV-DENV that encoded DENV-derived epitopes from HLA-A*02- and HLA-B*07-restricted NS and C virus proteins [30,34,52,53,54,55,57]. We focused on these two HLA alleles because they are carried by 50% and 25% of the European population, respectively [86], and are distributed in up to 30% of the population worldwide [87]. Hence, these selected epitopes represent the most physiologically relevant targets to be included in a first proof-of-concept vaccine candidate. Our findings demonstrated that ORFV-DENV primed specific and functional CD8^+^ T cell responses against each of the encoded DENV-derived peptides in experiments carried out in vitro. After exposure to ORFV-DENV, T cells from 67% (four of six) of the healthy donors responded simultaneously to 5 HLA-A*02- and HLA-B*07-restricted peptides. Furthermore, using ORFV-DENV in an in vitro restimulation assay, we observed broad and functional peptide-specific reactivation of memory CD8^+^ T cells in PBMCs from laboratory-confirmed DENV-positive donors. Specific responses were observed against both HLA-B*07-restricted peptides and the HLA-A*02-restricted peptide KLAEAIFKL. Exposure to ORFV-DENV did not result in the restimulation of peptide-specific CD8^+^ T cells against other HLA-A*02-restricted epitopes (i.e., VTLLCLIPTV and NIQTAINQV). This finding suggests that there may be no memory CD8^+^ T cells specific to these peptides in PBMCs from DENV-positive donors or that their frequencies were below the detection level of the assays used in this study. Both MS/MS [57] and HLA binding affinity predictions categorized NIQTAINQV as a weak binder. Additionally, multi-sequence alignment indicated that both VTLLCLIPTV and NIQTAINQV were less well-conserved within the virus family *Flaviviridae* compared to the HLA-B*07 epitopes. These results suggest that the most effective ORFV D1701-VrV vector vaccines will be those encoding highly conserved, HLA-restricted, and immunogenic viral epitopes. Our alignment revealed that both HLA-B*07-restricted epitopes were highly conserved; TV003/TV005 vaccine studies also demonstrated CD8^+^ T cell responses to these serotype-conserved epitopes [30]. The greater frequencies of T cell responses to HLA-B*07-restricted DENV-derived peptides in both HLA tetramer and ICS assays are consistent with recent hypotheses regarding the role of HLA in DENV disease pathogenesis. Weiskopf et al. [34] reported that specific HLA types correlate with the magnitude of CD8^+^ T cell responses as evaluated by differential cytokine production. For example, HLA-B*07 was found to induce protective and strong immune responses while HLA-A*24 was associated with a reduced number of CD8^+^ T cells and limited diversity of cytokine expression [88,89,90,91]. We also found that ORFV-DENV expressing DENV epitopes could restimulate memory CD8^+^ T cell responses in patients who had recovered from ZIKV infection. Specifically, we observed the restimulation of HLA-B*07-restricted NS3- and NS5-specific lymphocytes in PBMCs from one donor. In contrast, the least conserved HLA-A*02-restricted epitopes were unable to elicit peptide-specific T cell responses. Hence, ORFV-DENV represents a vaccine candidate that may induce and restimulate CD8^+^ T cell responses against a variety of peptide antigens that are conserved among the *Flaviviridae*. Several studies have already noted the importance of identifying conserved and cross-reactive T cell and B cell epitopes to prohibit nonspecific cellular and/or sub-neutralizing humoral responses to related viruses [92,93,94,95,96,97]. There is currently a large demand for protective and cross-protective vaccines, given the recent outbreaks of ZIKV in South America and the rise in severe outcomes, including neonatal ZIKV syndrome.

## 5. Conclusions

We have developed a novel and promising DENV vaccine candidate, ORFV-DENV, based on the ORFV D1701-VrV vector platform. We found that ORFV-DENV expressing both HLA-A*02- and HLA-B*07-restricted DENV antigens induces potent CD8^+^ T cell immune responses. These results serve as a proof-of-concept and support the development of ORFV-D1701-VrV-vectored vaccine candidates that encode immunogenic epitopes from additional flaviviruses. Using this approach, we have achieved several important goals, including the (i) identification and characterization of a direct immune response to cross-reactive, highly conserved, and immunogenic epitopes, (ii) the avoidance of nonspecific cellular and sub-neutralizing humoral responses that may enhance severe disease outcomes, and (iii) the identification of antigen-specific CD8+ T cell responses that are comparable to or higher than those induced by the current advanced vaccine candidates with (iv) an excellent safety profile [44]. Furthermore, protective immune responses against DENV might be enhanced by the simultaneous expression of DENV-derived B cell and CD4^+^ T cell epitopes.

## Figures and Tables

**Table 1:** DENV-derived peptides identified by the Immune Epitope Database. The HLA-A*02- or HLA-B*07-restricted peptides epitopes identified by this method were located in the DENV non-structural (NS) proteins 3, 4B, and 5 as well as the capsid (C) protein. The peptides indicated were described previously in studies on natural infection (NI) or vaccines (Vac), or based on MS/MS epitope predictions (MS/MS). The selected epitopes utilized in this study are indicated with an asterisk (*). NetMHC 4.0 and SYFPEITHI HLA binding affinity (Aff) estimation scores predict that 4 of the 5 peptides will be strong binders (SB) to HLA-A*02 or HLBA-B*07. Strong and weak binding (WB) peptides were defined as % rank < 0.5 and > 2, respectively.

Protein ID	Protein	Sites of Sequence Homology among Serotypes	Organism	HLA	SYFPEITHI Score	NetMHC % Rank/Aff (nM)	References	Association
**P1**	NS5_3054–3062_	KLAEAIFKL *	DENV 2	A*02:01, A*02:03, A*02:06, and A*02:17	31	0.01/3.72 nM (SB)	[53,55,57]	NI; Vac
**P2**	NS4B_3353–3361_	NIQTAINQV * NIQVAINQV NIQAAINQV	DENV 1–4 DENV 1 DENV 2	A*02:01	23	7.00/2833.78 nM (WB)	[57]	MS/MS
**P3**	C_102–111_	VTLLCLIPTV * ITLLCLIPTV VTLYLGVMV VTLVLVGIV VLNPYMPTV	DENV 4 DENV 4 DENV 1–2 DENV 2 DENV 3	A*02:01	30	0.01/4.01 nM (SB)	[57]	MS/MS; Vac
**P4**	NS3_1698–1707_	APTRVVAAEM * APTRVVASEM	DENV 2–4 DENV 1	B*07:02, B*35:01	19	0.05/11.26 nM (SB)	[30,34,52,54,55]	NI; Vac
**P5**	NS5_2881–2890_	TPRMCTREEF * KPRICTREEF RPRLCTREEF NPRLCTREEF	DENV 2 DENV 1 DENV 3 DENV 4	B*07:02, B*35:01, and B*53:01	19	0.04/10.20 nM (SB)	[30,34,54,55]	NI; Vac

**Table 2:** Summary of the results of DC-based in vitro priming of CD8+ T cells isolated from healthy blood donors.

Protein ID	Protein	Peptide Sequence	HLA	Ratio (%) of Donors Exhibiting Positive Priming Responses In Vitro	Frequency (%) ofHLA-Tetramer Positive CD8^+^ T Cells	Frequency (%) of Cytokine-Expressing Peptide-Specific CD8^+^ T Cells		
						TNF^+^/IFN^−^	TNF^+^/IFN^+^	TNF^−^/IFN^+^
**P1**	NS5_3054__–__3062_	KLAEAIFKL	HLA-A*02	1/5 (20%)	0%	1.12%	3.35%	0%
**P2**	NS4B_3353__–__3361_	NIQTAINQV	HLA-A*02	2/5 (40%)	0.05–1.21%	0%	0%	0%
**P3**	C_102__–__111_	VTLLCLIPTV	HLA-A*02	2/5 (40%)	0.01–0.36%	0–32.49%	0.21–25.1%	0–4.88%
**P4**	NS5_2881__–__2890_	TPRMCTREEF	HLA-B*07	1/4 (25%)	0.83%	3.50%	1.10%	0%
**P5**	NS3_1698__–__1707_	APTRVVAAEM	HLA-B*07	3/4 (75%)	1.5–5.2%	0–4.71%	0.64–10.6%	0.39–9.09%

**Table 3:** Demographic information for donors with laboratory-confirmed DENV or ZIKV. Blood samples from 18 individuals were analyzed and PBMCs from nine donors were identified as HLA-B*07- and/or HLA-A*02-positive. DENV serotypes were examined previously for one participant only.

ID	Gender	Age	Year of Infection	Virus/Serotype	HLA-A*02	HLA-B*07
DENV-006	F	59	2018	DENV/N.A.	+	+
DENV-008	F	42	2017	DENV/2	+	+
DENV-009	F	48	2017	DENV/N.A.	+	-
DENV-010	M	65	2015	DENV/N.A.	+	-
DENV-012	F	42	2015	DENV/N.A.	+	+
DENV-015	F	70	2016	DENV/N.A.	+	-
DENV-016	F	64	2018	DENV/N.A.	+	-
DENV-020	F	54	2016	ZIKV	+	-
DENV-021	M	39	2016	ZIKV	+	+

**Table 4:** Alignment of the encoded amino acid sequences of proteins encoded by DENV serotypes 2 and 4 with ZIKV strain MR766 revealed sequence conservation in the range of 44–100% for 4 of the 5 peptides as shown. HLA-B*07-restricted DENV-derived peptides (P4 and P5) were highly conserved compared to HLA-A*02-restricted peptides (P1, P2, and P3). Amino acids highlighted in red are sites of sequence divergence.

Protein ID	Protein	Source	GenBank ID	Sequence	Conservancy
**P1**	NS5_3054–3062_	DENV-2	AAA73185.1	KLAEAIFKL	44%
		ZIKV	AAV34151.1	TLALAVIKY	
**P2**	NS4B_3353–3361_	DENV-4	AAU89377.1	NIQTAINQV	44%
		ZIKV	AAV34151.1	NIKDTVNMV	
**P3**	C_102–111_	DENV-4	AAU89375.1	VTLLCLIPTV	0%
		ZIKV	AAV34151.1	KRRGADTSIG	
**P4**	NS5_2881–2890_	DENV-2	AAA73185.1	TPRMCTREEF	70%
		ZIKV	AAV34151.1	RPRVCTKEEF	
**P5**	NS3_1698–1707_	DENV-2	AAA73185.1	APTRVVAAEM	100%
		ZIKV	AAV34151.1	APTRVVAAEM	

## Data Availability

Not applicable.

## Supplementary Materials

The following are available online at https://www.mdpi.com/article/10.3390/biomedicines9121862/s1, Figure S1: (A) Genomic map of ORFV-ReCoA2/B7 and ORFV-PrCoA2/B7 depicting the arrangement of the minigenes encoding HLA-A*02- (red boxes) and HLA-B*07- (blue boxes) restricted epitopes controlled by the authentic early promoter Pvegf. The GFP gene used as a selection marker for virus purification is expressed under the control of the synthetic eP2 promoter. The 5′ and 3′ sequences homologous to the vegf-e locus of ORFV flanked the minigenes and the GFP sequence, enabling direct homologous recombination with the virus backbone. (B) The genetic homogeneity of novel recombinants was verified by PCR targeting the vegf-e locus and resulted in the predicted amplicon size for ORFV-ReCoA2/B7 (1637 bp) and ORFV-PrCoA2/B7 (1541 bp). (C) Insert-specific PCR revealed the predicted amplicon size for ORFV-ReCoA2/B7 (265 bp) and ORFV-PrCoA2/B7 (176 bp). Transfer plasmid pV-DENV-2-GFP was used as the positive control. DNA isolated from the parental ORFV V12-Cherry and non-infected cells served as the negative control. Figure S2: Effective DC-based in vitro priming was confirmed by HLA tetramer analysis and/or ICS. After the third restimulation, ICS was performed in cells challenged with a pool of DENV-derived synthetic peptides. After the fourth restimulation, HLA tetramer and ICS were performed using each peptide individually to reveal CD8^+^ T cell peptide specificity. (A) An example of HLA tetramer and ICS after a fourth restimulation with the peptide NIQTAINQV. ICS identified no cytokine-releasing T cells, although tetramer staining was positive with a frequency of 1.21%. (B) An example of CD8^+^ T cells primed with the peptide VTLLCLIPTV. No CD8^+^ T cells were detected by HLA tetramer staining, although 25% expressed TNF-α and IFN-γ by ICS. (C) Effective priming of CD8^+^ T cells with the HLA-B*07-restricted DENV NS3 peptide APTRVVAAEM. HLA tetramer staining and ICS for TNF-γ and IFN-γ detected positive CD8^+^ T cells with frequencies of 28.4% and 10.6%. Figure S3: After PBMC isolation from peripheral blood from DENV-confirmed participants, cells were stained with mouse-derived anti-human HLA-A*02 or anti-human HLA-B*07 antibodies. One unstained set of PBMCs per sample was included as a negative control. Flow cytometric evaluation identified nine participants who were either HLA-A*02- and HLA-B*07-positive, HLA-A*02-positive alone, or negative for both HLA class I types. Figure S4: Examples of HLA tetramer staining (A) and ICS (B) after the restimulation of PBMCs from a confirmed DENV-positive donor with ORFV-DENV (ORFV). Synthetic peptides and ORFV D1701-VrV expressing GFP only (mock) were used as positive and negative controls, respectively. Table S1: Selection of peptides encoded by ORFV D1701-VrV and provided as control peptides in the DC-based in vitro priming experiments featuring CD8^+^ T cells of healthy blood donors or in vitro restimulation of CD8^+^ T cells of DENV-confirmed participants. Four peptides were identified as strong HLA class I binders (SBs) within their respective HLA class I type by NetMHC 4.0 (% rank < 0.5). Aff, affinity; HIV-1, human immunodeficiency virus 1; HHV-5, human herpesvirus 5 (cytomegalovirus); HHV-4, human herpesvirus 4 (Epstein–Barr virus); IAV, influenza A virus. Table S2: Insert-specific primers for the identification of ORFV-DENV. Primer pairs for GFP and mcherry-specific PCRs as well as vegf-e-locus-specific primers, as previously described [44].
