# Supplementary figures and images for: A Novel Orf Virus D1701-VrV-Based Dengue Virus (DENV) Vaccine Candidate Expressing HLA-Specific T Cell Epitopes: A Proof-of-Concept Study
Source: Biomedicines. 2021 Dec 8;9(12):1862. doi: 10.3390/biomedicines9121862 (PMC8698572; doi:10.3390/biomedicines9121862)

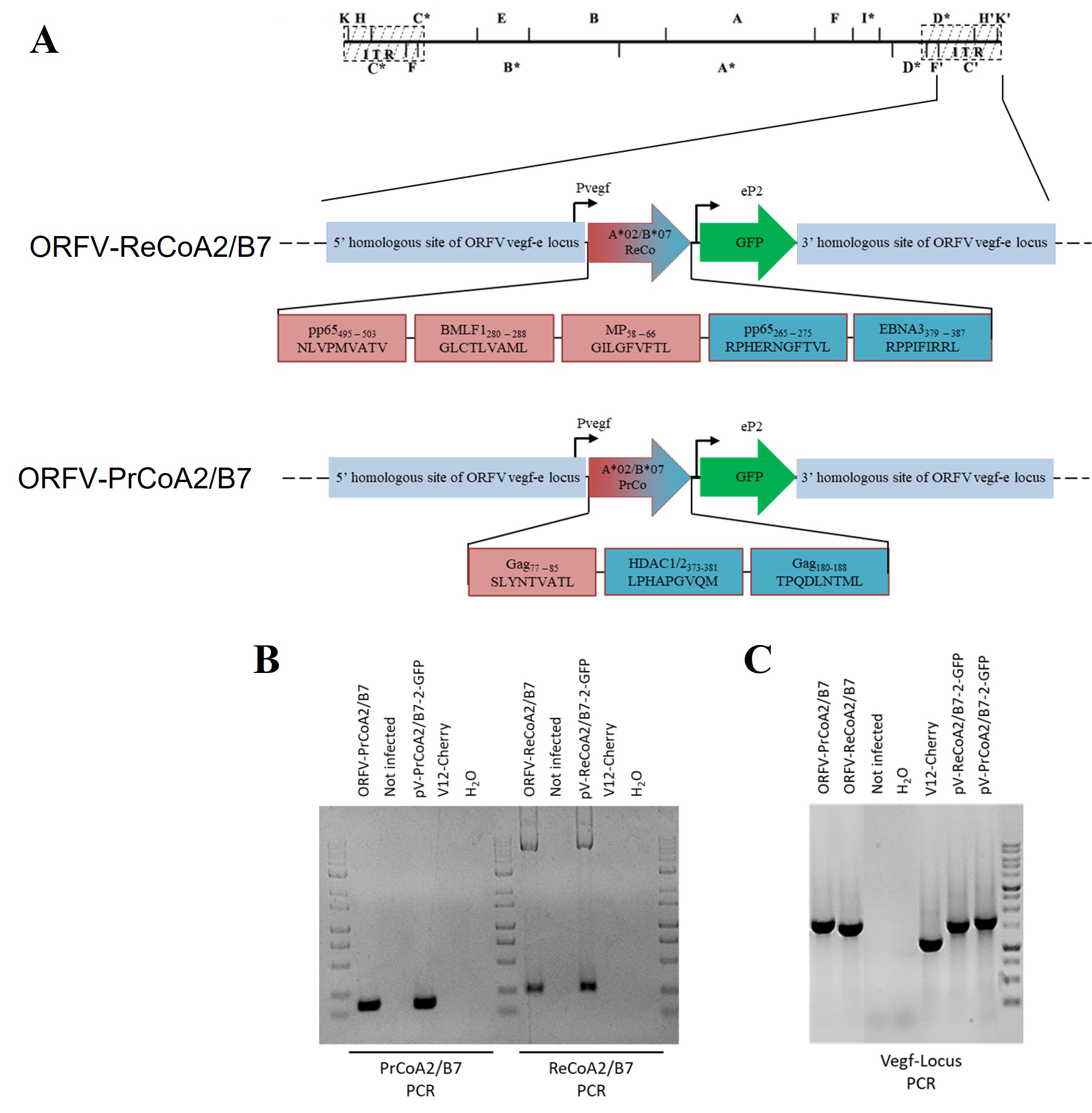

Supplement: Supplementary file 1 [file biomedicines-09-01862-s001.zip › Figure S1 new.jpg]

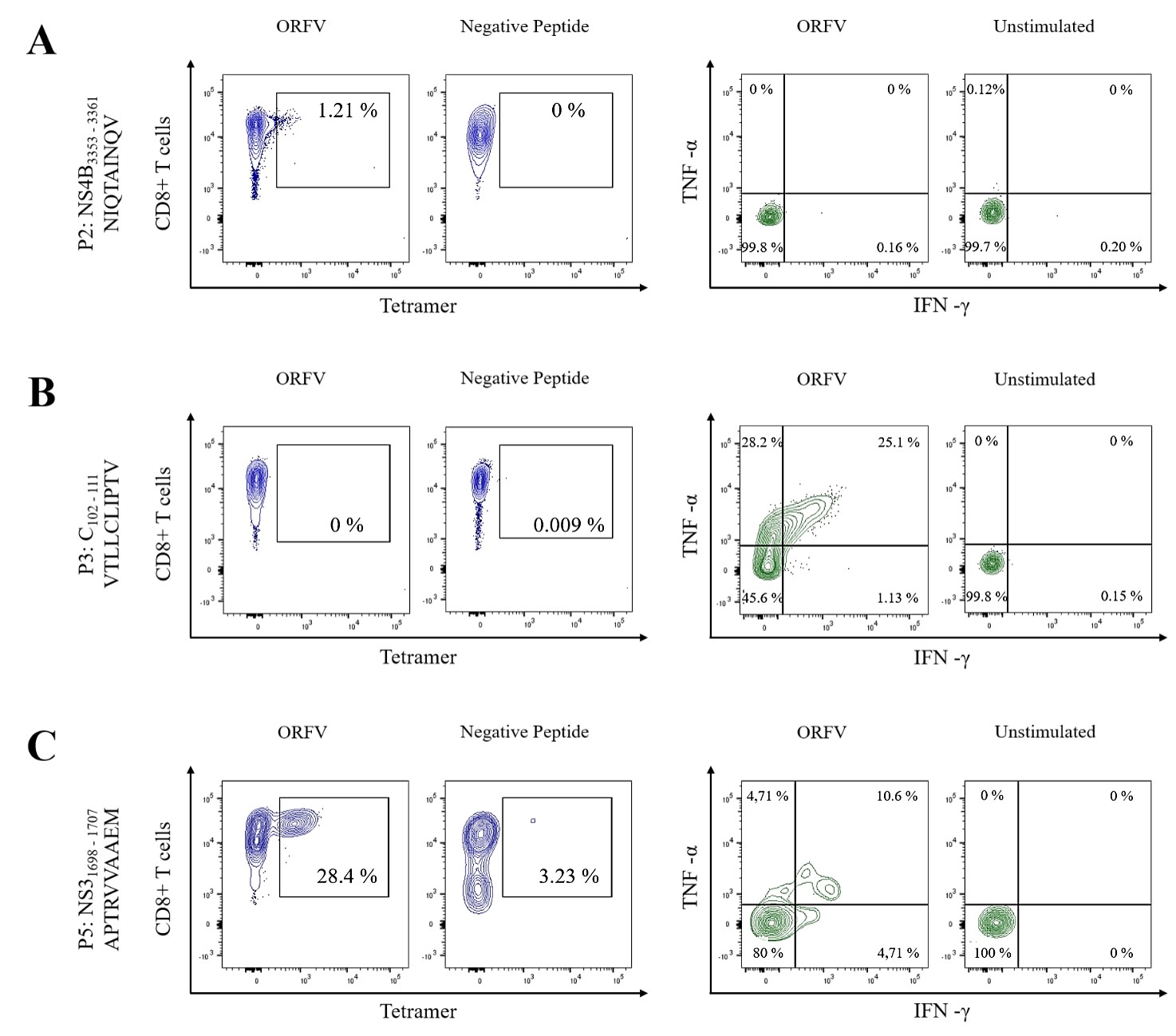

Supplement: Supplementary file 1 [file biomedicines-09-01862-s001.zip › Figure S2 new.jpg]

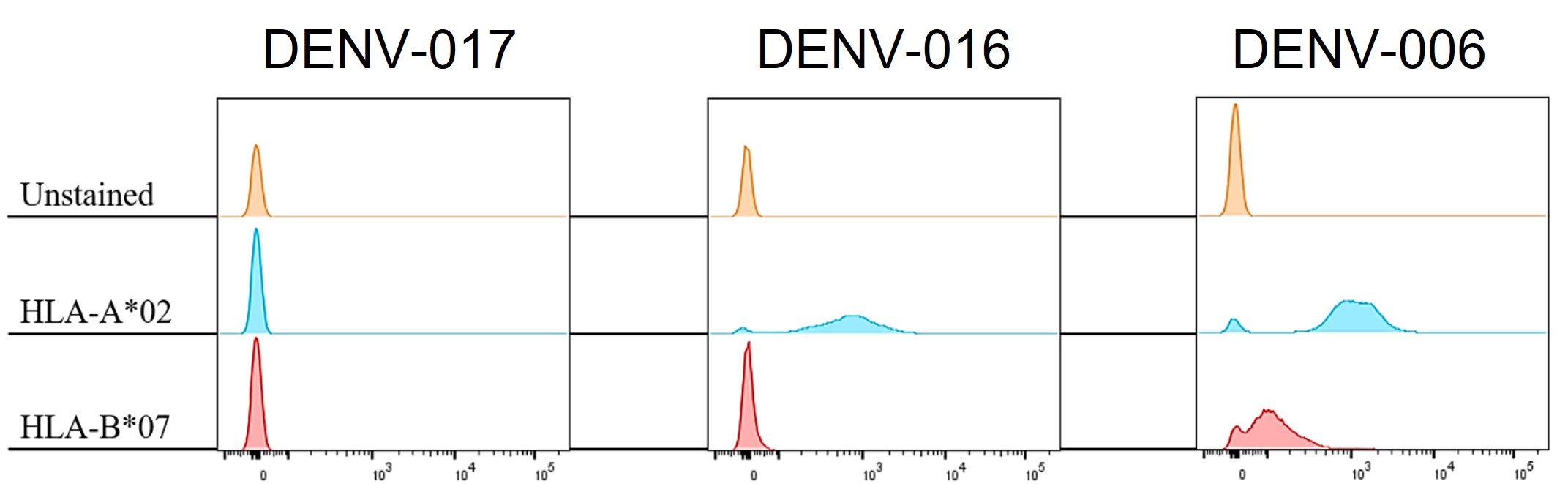

Supplement: Supplementary file 1 [file biomedicines-09-01862-s001.zip › Figure S3 new.jpg]

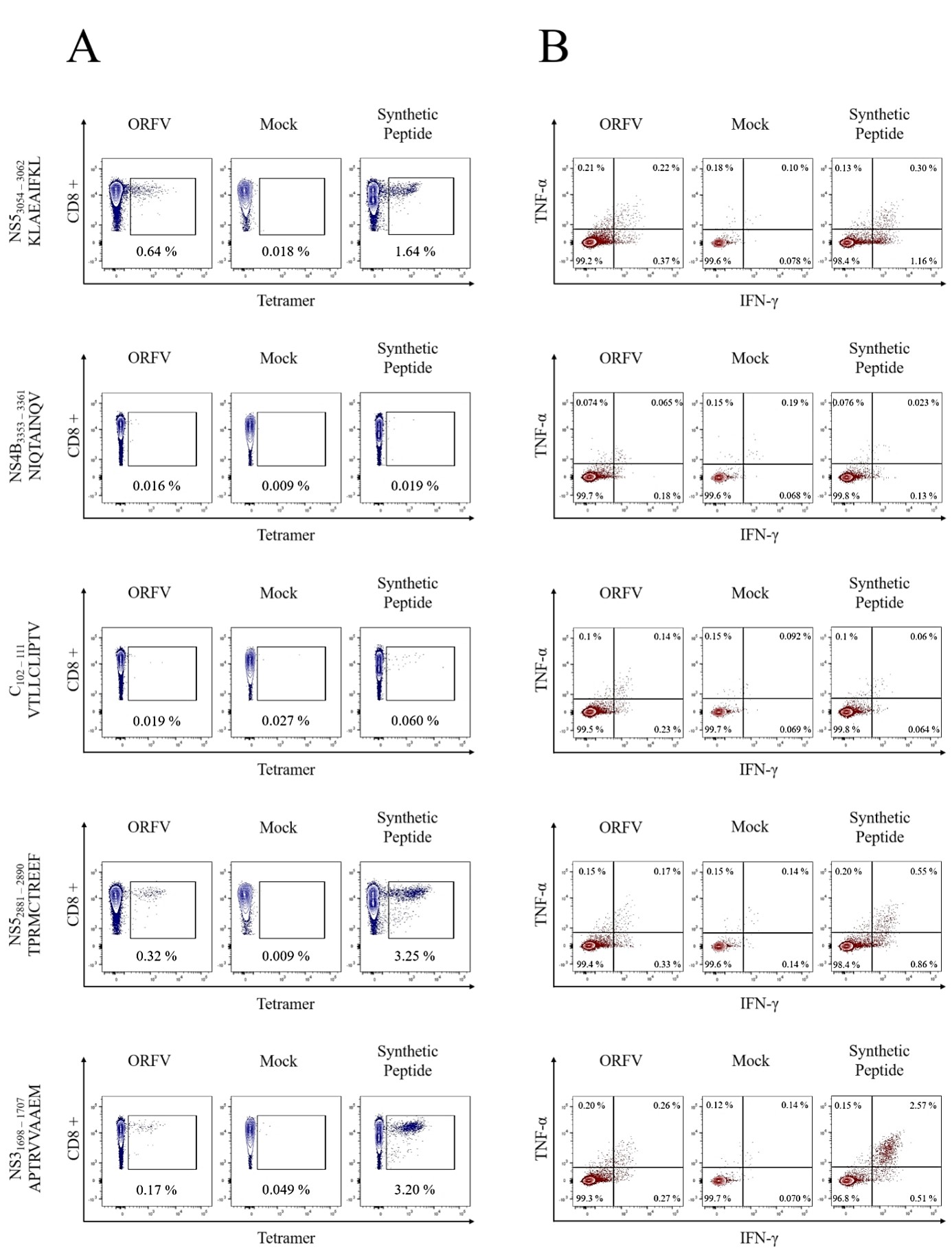

Supplement: Supplementary file 1 [file biomedicines-09-01862-s001.zip › Figure S4 new.jpg]

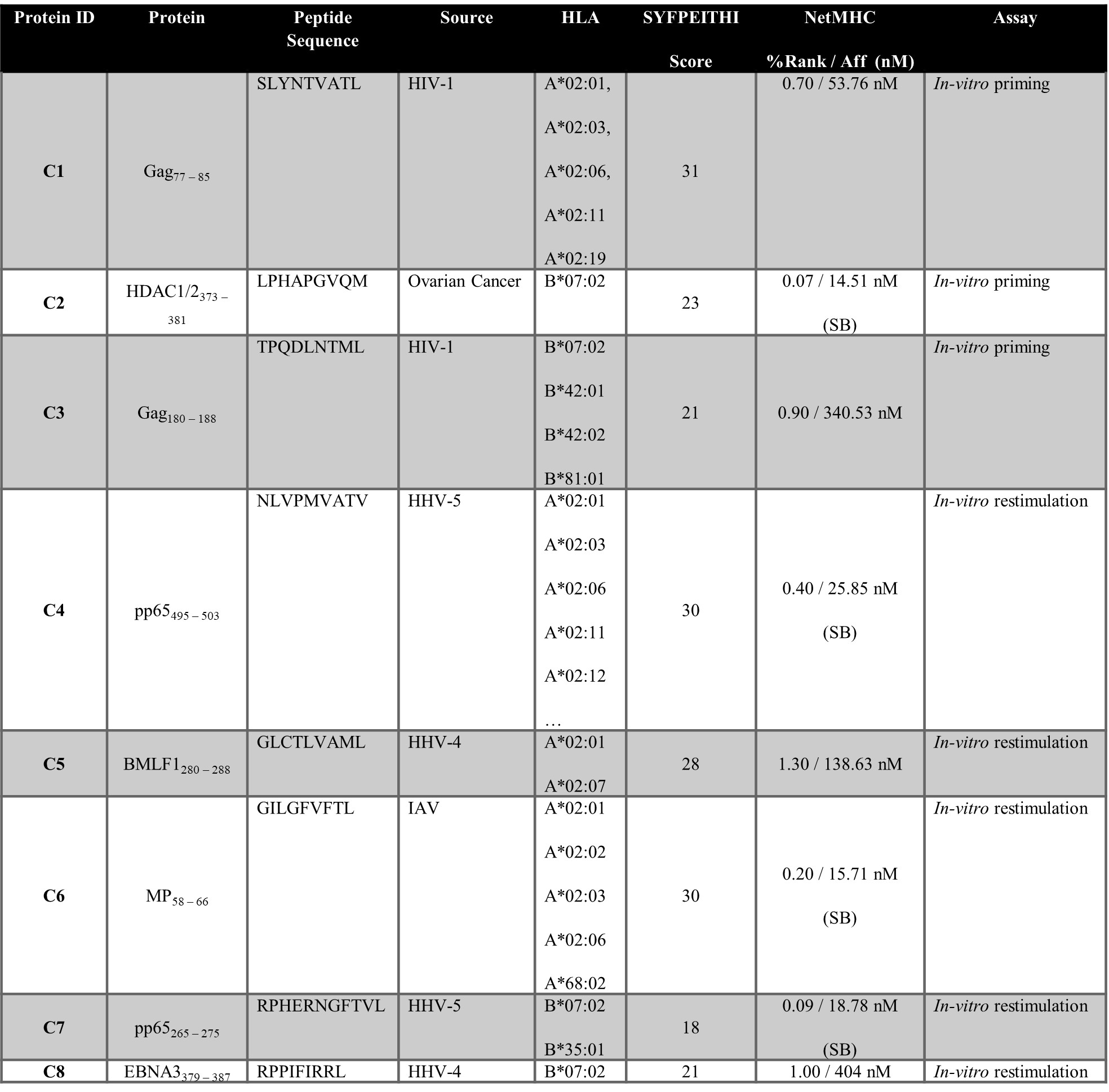

Supplement: Supplementary file 1 [file biomedicines-09-01862-s001.zip › Table S1 new.jpg]

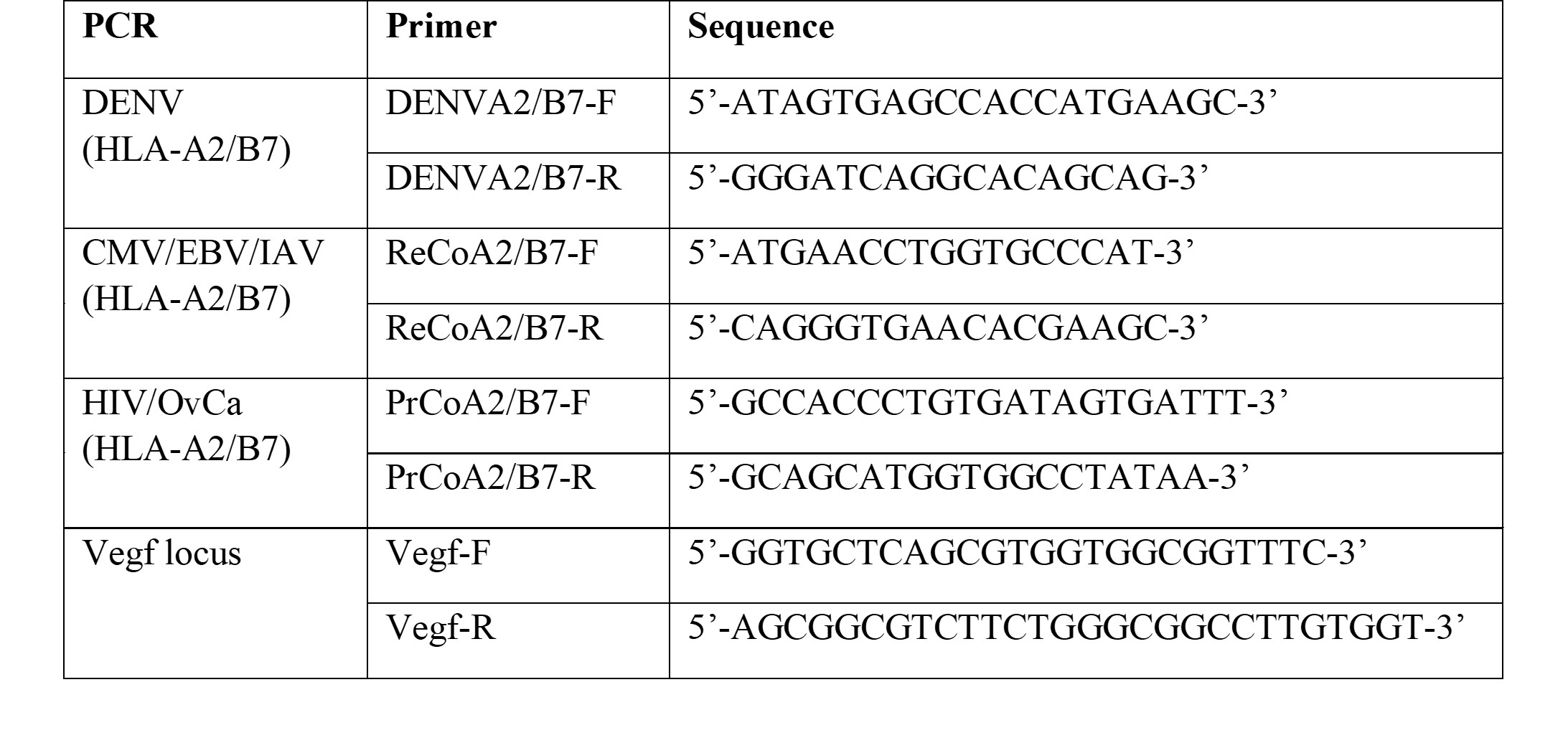

Supplement: Supplementary file 1 [file biomedicines-09-01862-s001.zip › Table S2 new .jpg]
